# Increased expression of the NLRP3 inflammasome components in patients with Behçet’s disease

**DOI:** 10.1186/s12950-015-0086-z

**Published:** 2015-07-02

**Authors:** En Hyung Kim, Mi-Jin Park, Sun Park, Eun-So Lee

**Affiliations:** Department of Dermatology, Ajou University School of Medicine, 164 Worldcup-ro, Yeongtong-Gu, Suwon, 443-380 South Korea; Department of Biomedical Sciences, The Graduate School, Ajou University and Department of Microbiology, Ajou University School of Medicine, Suwon, South Korea; Present address: Department of Dermatology, Cheil General Hospital and Women’s Healthcare Center, Dankook University College of Medicine, Cheonan, South Korea

**Keywords:** Behçet’s disease, Inflammasome, NLRP3, Interleukin-1β

## Abstract

**Background:**

Behçet’s disease (BD) is a systemic inflammatory disease with manifestations including recurrent oral and genital ulcerations, and vasculitis involving the skin, mucosa, joints, eyes, veins, arteries, nervous and gastrointestinal systems. BD is seen as a disease at the crossroad between autoimmune and autoinflammatory syndromes, possibly triggered by an aberrant response to infectious stimuli. The relevance of Gram negative bacteria-mediated oral inflammation with the increased expression of NACHT, LRR, and PYD domains-containing protein 3 (NLRP3), leading to systemic inflammation, prompted us to investigate the expression of NLRP3 inflammasome components and its link with IL-1β hypersecretion.

**Findings:**

When peripheral blood mononuclear cells (PBMCs) from 15 active, 15 stable BD patients and 15 healthy volunteers were stimulated, the basal and LPS-induced expressions of NLRP3 inflammasome components were significantly increased at both mRNA and protein levels in BD patients compared to healthy controls. Also, increased expression of NLRP3 and ASC was observed in 25 BD skin lesions compared to 25 erythema nodosum patients. Compatible with this, secretion of IL-1β by PBMCs stimulated with LPS alone or LPS plus ATP was increased in BD compared to healthy controls, which was suppressed by caspase-1 inhibitor.

**Conclusion:**

Our findings suggest the possible link between increased IL-1β secretion and increased expression of NLRP3 inflammasome components in BD patients with skin manifestations.

**Electronic supplementary material:**

The online version of this article (doi:10.1186/s12950-015-0086-z) contains supplementary material, which is available to authorized users.

## Introduction

Behçet’s disease (BD) is a systemic inflammatory disease with manifestations including recurrent oral and genital ulcerations, and vasculitis involving the skin, mucosa, joints, eyes, veins, arteries, nervous and gastrointestinal systems. BD is currently seen as a disease at the crossroad between autoimmune and autoinflammatory syndromes, possibly triggered by an aberrant response to infectious stimuli [1]. Oral microbial flora have long been implicated in the pathogenesis of BD [2]. However, it is not clear how infection induces an immune response and initiates the development of BD. Recently it was shown that NACHT, LRR, and PYD domains-containing protein 3 (NLRP3) inflammasome is upregulated when infected by Porphyromonas gingivalis, a G(−) bacteria, and in some cases lead to induction and sustained aortic or gingival inflammation [3]. Also, peptidoglycan and LPS induced IL-1β via the TLR2/4 and reactive oxygen species–NLRP3 inflammasome-dependent pathways in ocular BD [4]. As oral ulcer precede BD for many years and EN like lesions, one of most common skin manifestions in BD, which are characterized by septal and lobular panniculitis and most importantly vasculitis, we investigated whether NLRP3 inflammasome expression is increased at the mRNA and protein levels in BD patients with skin manifestations. Also, we studied whether IL-1β production was mediated through NLRP3 inflammasome dependent pathway in BD.

## Methods

### Patients

Blood samples were taken from 15 active, 15 stable BD patients and 15 healthy volunteers (HC) (Table 1). All patients consisted of BD patients who presented themselves for the first time or were monitored at the Department of Dermatology, Ajou University Hospital. BD patients met the Diagnostic criteria of the BD Research Committee of Japan. The active group patients had at least one of the BD symptoms despite the treatment and inactive group patients were in well-controlled states. Informed consent was obtained prior to the study. This study was approved by the Institutional Review Board (IRB no.: AJIRB-GN3-07-098, AJIRB-GGEN-GEN-10-119).

**Table 1:** Characteristics of Behçet’s disease (BD) patients and healthy controls

Demographics	Active BD	Inactive BD	Healthy control
Age (mean ± SD)	41.86 ± 7.54	43.6 ± 8.04	38.93 ± 6.94
Sex (male:female)	6:9	7:8	7:8
Clinical characteristics of BD patients	Case number (%)		
Major symptoms			
Oral ulcer	15 (100)		
Genital ulcer	10 (67)		
Skin lesion	10 (67)		
Ocular lesion	4 (27)		
Minor symptoms			
Arthritis	2 (13)		
GI involvement	1 (7)		
Epididymitis	0		
Cardiovascular	1 (7)		
CNS involvement	1 (7)		
Medication			
Immunosuppressive agent^a^	6 (40)	0 (0)	
Anti-inflammatory drugs^b^	9 (60)	7 (46)	

^a^Methylprednisolone 8–24 mg/d

^b^Colchicine 1.2 mg/d ± minocycline 100 mg/d ± pentoxiphylline 800 mg/d ± sulfasalazine 1000 mg/d

### Immunohistochemistry

Six mm punch skin biopsies of erythema nodosum (EN)-like lesions of 25 BD and 25 EN patients were performed. Formalin-fixed and paraffin-embedded tissues of EN-like lesions in BD were cut (3-μm thickness) and mounted onto slides. Specimens were deparaffinated and endogenous peroxidase activity was blocked by 3 % H_2_O_2_ in methanol for 15 min at room temperature. After rinsing in phosphate-buffered saline (PBS) for 10 min, the nonspecific binding sites were blocked by blocking solution for 10 min at room temperature and all specimens were incubated with polyclonal antibodies against NLRP3 (1:50 dilution, mouse, Alexis Biochemicals, San diego, CA, USA) and ASC (1:100 dilution, rabbit, Lifespan bioscience, Seattle, WA) for 30 min at room temperature. Next, HRP polymer (Thermo scientific, Fremont, CA, USA) was applied and incubated for 30 min at room temperature. After washing in PBS for 10 min, bound antibodies were visualized by incubation with AEC chromogen system (Thermo scientific, Fremont, CA, USA). Slides were counterstained with hematoxylin. Negative controls were isotype matched. The image was analyzed using Image Pro Plus Version 4.5 (Media Cybertics Co., MD, U.S.A.) (Additional file 1).

### Quantitative real time PCR

Peripheral blood mononuclear cells (PBMCs) were prepared from heparinized blood samples by Ficoll Hypaque density gradients (Ficoll paque^TIM^ plus, StemCell Technologies, Vancouver, BC, Canada). PBMCs were stimulated for 4 h with 100 ng/ml lipopolysaccharide (LPS; Sigma-Aldrich). After 4 h, RPMI containing 1 mM adenosine 5-triphosphate (ATP; Sigma-Aldrich) was added to the cells for another 15 min (LPS/ATP). In separate experiments, 20 μM zYVAD(Ome)-FMK an irreversible caspase-1 inhibitor (CaspI; Enzo life science, PlymouthMeeting, PA) was added. The method for Quantitative real time PCR is described in Additional file 2.

### Western blotting

Briefly, freshly isolated PBMCs and stimulated PBMCs were harvested and lysed in RIPA buffer (Sigma–Aldrich, St. Louis, MO, USA) containing protease inhibitors. Cell extracts were run on Bolt™ 4–12 % Bis–Tris Plus Gel (life technologies, Carlsbad, CA, USA) and transferred onto polyvinylidene difluoride membranes (Merck Milipore, Darmstadt, Germany). Following transfer, the membrane was blocked overnight at room temperature with PBS/0.2 % Tween-20/5 % skim milk. Blots were incubated with primary antibodies anti-NLRP3, anti-apoptosis-associated speck-like protein containing a CARD (ASC), and anti-caspase-1 antibodies (Abcam, Cambridge, MA). The membranes were incubated in a solution containing an appropriate secondary Ab (either anti-rabbit IgG or anti-mouse IgG Ab) linked to horseradish peroxidase (Invitrogen). Bands were visualized with Immobilon Western Chemiluminescent HRP Substrate (Merck Milipore, Darmstadt, Germany) (Additional file 2).

### ELISA for IL-1β

Total IL-1β and mature IL-1β level in the supernatants was measured with a commercial ELISA kit from R&D Systems according to the manufacturer’s protocols.

### Statistics

The data are presented as mean ± standard deviation (S.D.). Data were analyzed by one-way analysis of variance followed by the Scheffé test for overall multiple comparisons among HC, stable BD, active BD. Student’s *t*-test was used for comparison between two groups. SPSS 17.0 (SPSS Inc., Chicago, IL) was used. A *p*-value <0.05 was considered to indicate statistical significance.

## Results and discussion

**Fig. 1:**
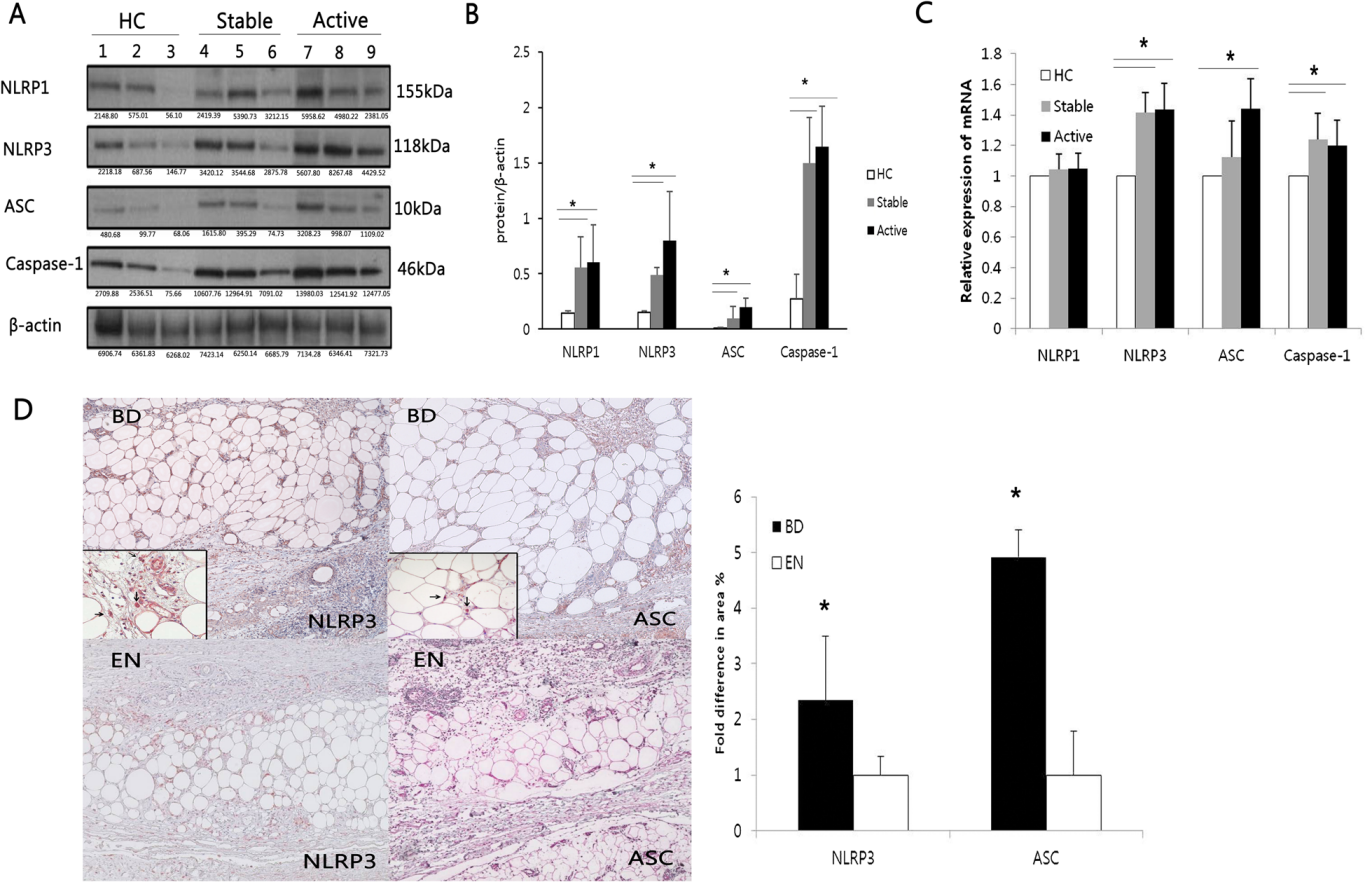
The protein and mRNA expression of NLRP3, ASC, caspase-1 is increased in Behçet’s disease (BD). **a** and **b** Representative western blot analysis (*lane 1*–*3*: healthy control, *lane 4*–*6*: stable BD, *lane 7*–*9*: active BD) and quantitation of NLRP1, NLRP3, ASC and caspase-1 from cell lysates of freshly isolated PBMCs. β-actin was used as loading control (*n* = 5 per group). **c** The mRNA expression of NLRP1, NLRP3, ASC and caspase-1 was measured from freshly isolated PBMCs by real time PCR and normalized against the expression levels of glyceraldehyde 3-phosphate-dehydrogenase. The relative values are shown as a fold change to HC with no treatment (*n* = 8 per group). **d** Immunohistochemistry staining of skin lesions of BD patients and control EN lesions with anti-NLRP3 (*left*) and anti-ASC (*right*) antibody showed NLRP3 and ASC expression in inflammatory cells infiltrating the subcutaneous tissue (×200). Computer assisted image analysis showed significant increase in NLRP3 and ASC stained area in BD patients. The values are shown as a fold change to EN. The *smaller box* is a magnified region (×400) (*n* = 25 per group). Data are represented as mean ± S.D. (*p < 0.05), (−) no treatment. NLRP3: NACHT, LRR, and PYD domains-containing protein 3; PBMCs: peripheral blood mononuclear cells; HC: healthy volunteers; EN: erythema nodosum

Previous reports show NLRP3 inflammasome expression is increased in inflammatory diseases [5]. We investigated the protein and mRNA levels of the different components of NLRP3 inflammasomes, and NLRP1 for comparison. The protein expression of NLRP3 inflammasome components in PBMCs of BD patients was analyzed by western blotting (Fig. 1a and b). The mean values of normalized NLRP3, ASC and caspase-1 protein levels were significantly up-regulated in active and stable BD compared with HC. NLRP3, ASC and caspase-1 mRNA expression was significantly up-regulated in active and stable BD compared with HC (Fig. 1c). Next, we examined skin lesions for in situ expression of NLRP3 and ASC to correlate our in vitro findings. NLRP3 and ASC in skin lesions of BD and control EN patients were detected by immunohistochemistry. NLRP3 and ASC was mostly expressed by CD68+ macrophages/monocytes (Additional file 3). Image analysis showed that NLRP3 and ASC expression was significantly increased in BD skin lesions (Fig. 1d). These findings show that mRNA and protein levels of NLRP3 inflammasome components are increased in BD patients.

**Fig. 2:**
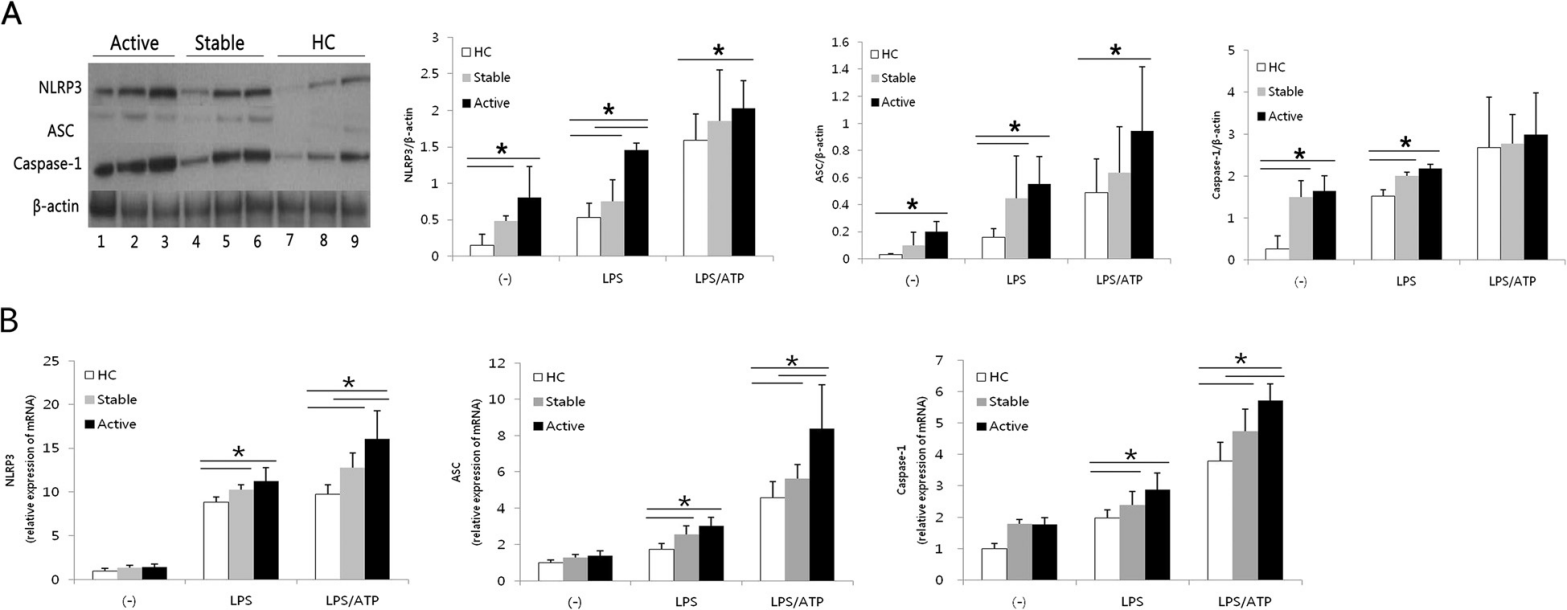
The induced expression of NLRP3, ASC and caspase-1 is increased in Behçet’s disease (BD). PBMCs were initially stimulated for 4 h with LPS (100 ng/ml). After 4 h, ATP (1 mM) was added to the cells for another 15 min (LPS/ATP). **a** Representative western blot analysis and quantitation of NLRP3, ASC and caspase-1 from cell lysates of stimulated PBMCs. β-actin was used as loading control. (*lane 1*,*4*,*7*: no treatment, *lane 2*,*5*,*8*: LPS, *lane 3*,*6*,*9*: LPS/ATP) (*n* = 5 per group). **b** The mRNA expression of NLRP3, ASC and caspase-1 was measured by real time quantitative RT-PCR and normalized against the expression levels of glyceraldehyde 3-phosphate-dehydrogenase. The relative values are shown as a fold change to HC with no treatment (*n* = 8 per group). Data are represented as mean ± S.D. (*p < 0.05), (−) no treatment. NLRP3: NACHT, LRR, and PYD domains-containing protein 3; PBMCs: peripheral blood mononuclear cells; LPS: lipopolysaccharide; ATP: adenosine 5-triphosphate; HC: healthy volunteers

Toll-like receptor signaling induces transcription of NLRP3 and IL-1β [6, 7]. NLRP3 inflammasome is activated by canonical stimuli like ATP or Nigericin and noncanonical stimuli like live gram negative bacteria [8]. Therefore, we checked whether LPS alone, or ATP stimulation after LPS priming (LPS/ATP), affected the expression of NLRP3 inflammasome components in PBMCs of BD patients. The protein levels of, NLRP3, ASC and caspase-1 were higher following LPS stimulation compared to no stimulation in all the groups and the levels increased significantly in active and stable BD compared to HC (Fig. 2a). Following LPS/ATP stimulation, NLRP3 and ASC protein levels were significantly up-regulated only in active BD compared to HC (Fig. 2a). The mRNA levels of, NLRP3, ASC and caspase-1 were higher after LPS/ATP stimulation compared to single stimulus of LPS in all the groups. Furthermore, they were significantly increased in the presence of LPS or LPS/ATP in active and stable BD compared to HC (Fig. 2b). These findings show that LPS/ATP stimulation resulted in significantly higher expression of NLRP3 inflammasome component at protein and mRNA levels in PBMCs of BD patients.

**Fig. 3:**
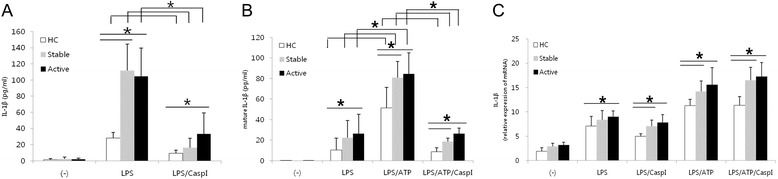
Caspase-1 inhibits increase of IL-1β secretion by peripheral blood mononuclear cells (PBMCs) following NLRP3 activation. PBMCs were initially stimulated for 4 h with LPS (100 ng/ml) with or without 20 μM zYVAD(Ome)-FMK, an irreversible caspase-1 inhibitor (LPS/CaspI). After 4 h, ATP (1 mM) was added to the cells for another 15 min (LPS/ATP or LPS/ATP/CaspI). **a** Total IL-1β (*n* = 15 per group) and **b** mature processed IL-1β (*n* = 9 per group) was quantitated in the supernatant of stimulated PBMCs by ELISA. **c** The mRNA expression of IL-1β was measured by real time PCR and normalized against the expression levels of glyceraldehyde 3-phosphate-dehydrogenase. The relative values are shown as a fold change to HC with no treatment (*n* = 8 per group). Data are represented as mean ± S.D. (*p < 0.05), (−) no treatment. PBMCs: peripheral blood mononuclear cells; NLRP3: NACHT, LRR, and PYD domains-containing protein 3; LPS: lipopolysaccharide; ATP: adenosine 5-triphosphate

To ascertain whether the increased NLRP3 inflammasome components might contribute to increased secretion of IL-1β in BD, we assessed IL-1β secretion by PBMCs stimulated with LPS or LPS/ATP (Fig. 3a and b) In accordance with previous reports [7] showing that peripheral blood monocytes stimulated with LPS release ATP and consequently secrete IL-1β, treatment of PBMCs with LPS alone increased IL-1β secretion compared to no stimulation. This effect was suppressed by caspase-1 inhibition and significantly higher in BD compared to HC (Fig. 3a). Additionally, mature IL-1β secretion in the presence of LPS/ATP was significantly higher in active and stable BD than HC and suppressed by caspase-1 inhibitor (Fig. 3b). There were significant differences in LPS-induced and LPS/ATP-induced IL-1β mRNA levels between BD and HC (Fig. 3c). However, caspase-1 inhibitor suppressed mature IL-1β secretion in the presence of LPS/ATP without a decrease in mRNA levels (Fig. 3b and c). These findings suggest that stimulation induced IL-1β expression and higher expression of NLRP3 inflammasome components in BD might contribute to increased IL-1β secretion in BD patients.

The hypothesis of an environmental trigger in BD patients with genetic susceptibilities has long been advocated [2]. These triggers range from infections to molecular mimicry such as heat-shock proteins (HSP), which are synthesized under cellular stress. Inflammasomes are involved in both pathways of pathogen and danger signal sensing, making it a good target for investigation in BD pathogenesis. Presence of a prolonged inflammation such as non-specific (pathergy) or urate induced skin responses suggests that innate and adaptive pathways are more integrated in BD [9]. The augmented adaptive responses can be the result of persistant oral and skin infections. Given the high frequency of oral ulcer and the decrease of some symptoms with minocycline, oral flora has been implicated in the pathogenesis of BD [10, 11]. Recent studies have linked oral infection to inflammasomes leading to sustained inflammation in atherosclerosis [3]. In this study we showed that the mRNA and protein levels of the NLRP3 inflammasome were upregulated in PBMCs of BD patients and this was further supported by in situ findings in skin lesions. This may be a coincidental finding due to tissue damage and release of DAMP, however the increase was more significant compare to RAU, which also shows tissue damage (Additional file 4). Thus increase in NLRP3 inflammasomes may have a role in BD pathogenesis.

Pathways of inflammasome and pathogen-associated molecular pattern such as toll-like receptors (TLRs) intersect as both are sensors of bacterial products. We have previously shown that TLR2 expression increases at both the mRNA and protein levels, along with TLR4 expression at the mRNA level in active BD patients [12]. LPS from microorganisms could initiate IL-1β synthesis process through TLRs and the inflammasomes. The extracellular ATP released from stressed or infected cells in oral tissue may further activate NLRP3 inflammasomes leading to IL-1β maturation and release. The released IL-1β can activate the release of other proinflammatory cytokines. In this study we showed that the components of the NLRP3 inflammasome pathway along with IL-1β mRNA level were higher after LPS/ATP challenge compared to LPS stimulation only in PBMCs of BD patients. The upregulation of components of the NLRP3 inflammasome pathway along with IL-1β mRNA corresponded with increased secretion of IL-1β. Also, IL-1β decreased when caspase-1 inhibitor was added.

IL-1β synthesis, maturation, and secretion are tightly regulated by Toll-like receptor (TLR) signaling and inflammasome activation [13]. IL-1β has significant role in BD pathomechanism. Specific IL-1β gene polymorphisms cause increased susceptibility to BD and IL-1β blockade with recombinant anti-IL-1β antibody gevokizumab has been used for BD treatment [14]. Thalidomide, an anti-inflammatory drug, used to treat BD, was found to decrease IL-1β through caspase-1 inhibition [15]. Therefore understanding the mechanism of NLRP3 in BD will not only help to elucidate their role in host responses to infectious agents and to danger signals, but may also contribute to the development of novel treatment of BD.

## Conclusion

In this study, we showed that expression of NLRP3 inflammasome components were increased in BD PBMCs and skin lesions. Also, IL-1β secretion was increased in BD PBMCs when NLRP3 inflammasome components were elevated and suppressed when caspase-1 inhibitor was added. Our findings suggest the possible link between increased IL-1β secretion and increased expression of NLRP3 inflammasome components in BD patients with skin manifestations.

## Additional files

Additional file 1:
**Image analysis of immunohistochemical staining.** Image signals were recorded on a personal computer and evaluated using Image Pro Plus Version 4.5 (Media Cybernetics Co., Silver Spring, MD, USA). For each staining, we established a standard for antibody (Ab) positivity and applied the same standards to the samples. The stained area per total area was measured and the ratio of positive Ab area (yellow) to the septal and lobular panniculitis area (green) was calculated. Each measurement was evaluated under constant magnification (×200). As inflammation presents mainly in and around the septa, fat lobules were left out when calculating the area. The image analysis was performed on a representative area of each specimen and repeated three times by three examiners and the mean was used for evaluation. Finally the data was expressed as fold difference between EN-like lesion and EN. Additional file 2:
**Detailed method for Quantitative real time PCR and Western blotting.**
Additional file 3:
**Erythema nodosum-like lesions are infiltrated with CD3+ T lymphocytes, CD68+ monocytes and macrophages, and Myeloperoxidase (MPO) + neutrophils.** Immunohistologic staining was performed on formalin-fixed and paraffin-embedded tissues of EN-like lesions with a panel of antibodies directed against mononuclear cell antigens using an indirect avidin-biotin immunoperoxidase technique. The degree of inflammatory cell infiltration, which also showed positivity to NLRP3 in serial section, were graded on a semi-quantitative scale of 0–4: 0, absent; 1, minimal; 2, mild; 3, moderate; 4, marked staining. All slides were evaluated by three examiners in a blinded fashion and the average score was calculated for each section. (A) anti-CD3 (1:200 dilution, mouse, Novocastra, Newcastle, UK), (B) anti-CD20 (1:300 dilution, mouse, Dako, Denmark), (C) anti-CD68 (1:80 dilution, mouse, Novocastra, Newcastle, UK), (D) anti-MPO (1:600 dilution, rabbit, NeoMarker, Fremount, CA, USA) antibody (×200) (*n* = 25). The semi-quantitative analysis of inflammatory cell infiltration was as follows; CD3+ T lymphocytes: 2.01 ± 1.11, CD20+ B lymphocytes: 0.75 ± 1.05, CD68+ monocytes/macrophages: 3.14 ± 0.65, MPO+ neutrophils: 2.69 ± 1.27. Data are represented as mean ± S.D. Additional file 4:
**The induced expression of NLRP3, ASC, caspase-1 and IL-1β is increased in Behçet’s disease (BD) compared to healthy control or recurrent apthous ulcer patients (RAU).** PBMCs were initially stimulated for 4 h with LPS (100 ng/ml) with or without 20 μM zYVAD(Ome)-FMK, an irreversible caspase-1 inhibitor (LPS/CaspI). After 4 h, ATP (1 mM) was added to the cells for another 15 min (LPS/ATP or LPS/ATP/CaspI). (A-D) The mRNA expression of NLRP3, ASC, caspase-1 and IL-1β was measured by real time quantitative RT-PCR and normalized against the expression levels of glyceraldehyde 3-phosphate-dehydrogenase. The relative values are shown as a fold change to HC with no treatment (*n* = 8 per group). (E) Total IL-1β (*n* = 15 per group) was quantitated in the supernatant of stimulated PBMCs by ELISA. Data are represented as mean ± S.D. (**p* < 0.05), (−): no treatment; LPS: lipopolysaccharide; ATP: adenosine 5-triphosphate; CaspI: caspase-1 inhibitor; HC: healthy volunteers, RAU: Recurrent apthous ulcer (disease control).

## Abbreviations

ATP: Adenosine 5-triphosphate
BD: Behçet’s disease
EN: Erythema nodosum
HC: Healthy volunteers
LPS: Lipopolysaccharide
RAU: Recurrent apthous ulcer
NLRP3: NACHT, LRR, and PYD domains-containing protein 3
PBMCs: Peripheral blood mononuclear cells
TLRs: Toll-like receptors
