# Supplementary figures and images for: Increased expression of the NLRP3 inflammasome components in patients with Behçet’s disease
Source: J Inflamm (Lond). 2015 Jul 2;12:41. doi: 10.1186/s12950-015-0086-z (PMC4487834; doi:10.1186/s12950-015-0086-z)

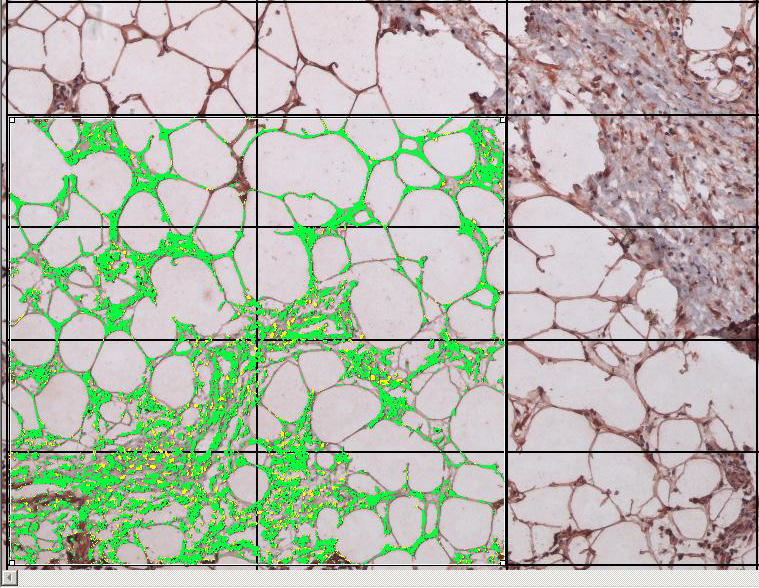

Supplement: Additional file 1: — Image analysis of immunohistochemical staining. Image signals were recorded on a personal computer and evaluated using Image Pro Plus Version 4.5 (Media Cybernetics Co., Silver Spring, MD, USA). For each staining, we established a standard for antibody (Ab) positivity and applied the same standards to the samples. The stained area per total area was measured and the ratio of positive Ab area (yellow) to the septal and lobular panniculitis area (green) was calculated. Each measurement was evaluated under constant magnification (×200). As inflammation presents mainly in and around the septa, fat lobules were left out when calculating the area. The image analysis was performed on a representative area of each specimen and repeated three times by three examiners and the mean was used for evaluation. Finally the data was expressed as fold difference between EN-like lesion and EN. [file 12950_2015_86_MOESM1_ESM.jpeg]

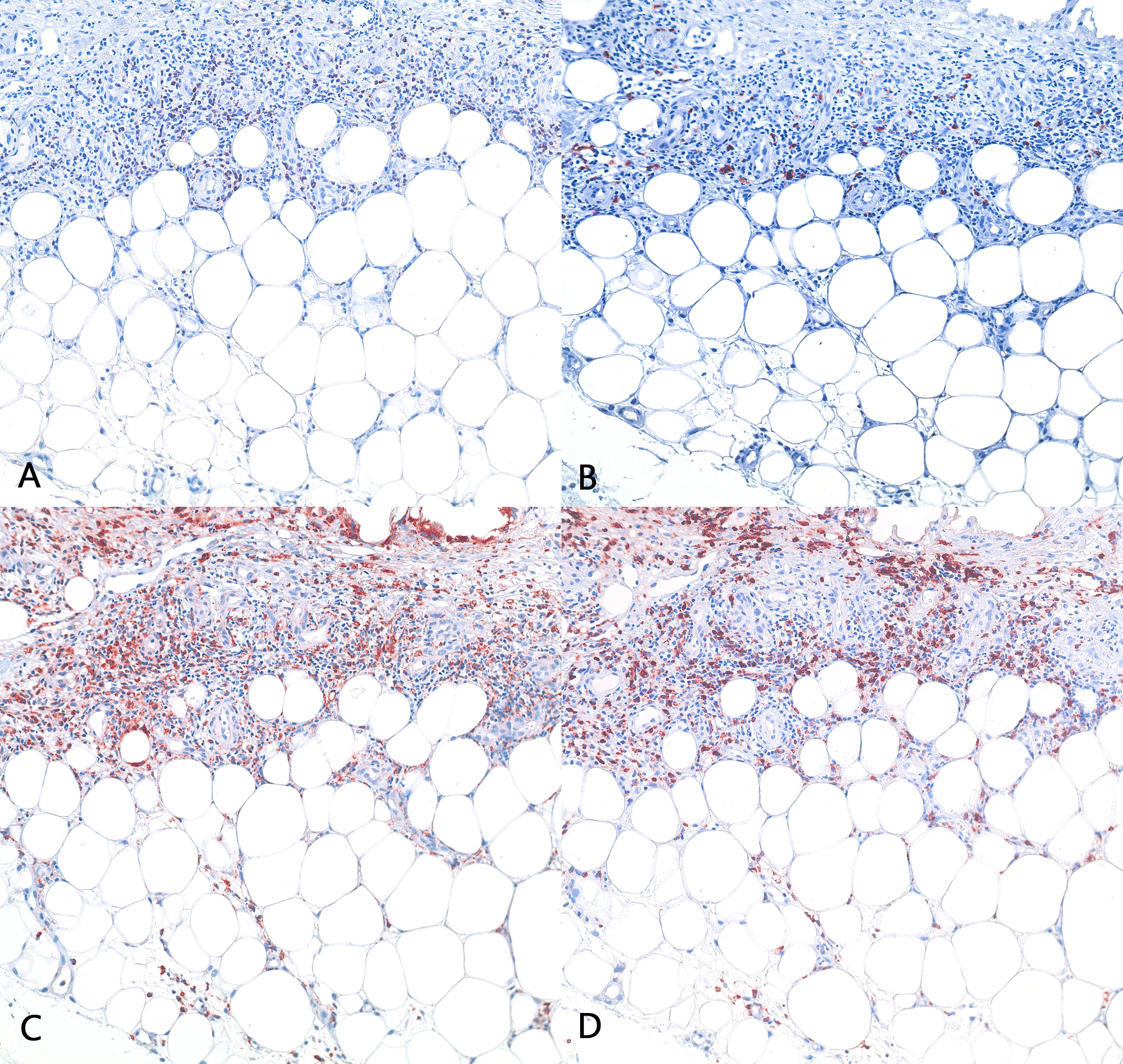

Supplement: Additional file 3: — Erythema nodosum-like lesions are infiltrated with CD3+ T lymphocytes, CD68+ monocytes and macrophages, and Myeloperoxidase (MPO) + neutrophils. Immunohistologic staining was performed on formalin-fixed and paraffin-embedded tissues of EN-like lesions with a panel of antibodies directed against mononuclear cell antigens using an indirect avidin-biotin immunoperoxidase technique. The degree of inflammatory cell infiltration, which also showed positivity to NLRP3 in serial section, were graded on a semi-quantitative scale of 0–4: 0, absent; 1, minimal; 2, mild; 3, moderate; 4, marked staining. All slides were evaluated by three examiners in a blinded fashion and the average score was calculated for each section. (A) anti-CD3 (1:200 dilution, mouse, Novocastra, Newcastle, UK), (B) anti-CD20 (1:300 dilution, mouse, Dako, Denmark), (C) anti-CD68 (1:80 dilution, mouse, Novocastra, Newcastle, UK), (D) anti-MPO (1:600 dilution, rabbit, NeoMarker, Fremount, CA, USA) antibody (×200) (n = 25). The semi-quantitative analysis of inflammatory cell infiltration was as follows; CD3+ T lymphocytes: 2.01 ± 1.11, CD20+ B lymphocytes: 0.75 ± 1.05, CD68+ monocytes/macrophages: 3.14 ± 0.65, MPO+ neutrophils: 2.69 ± 1.27. Data are represented as mean ± S.D. [file 12950_2015_86_MOESM3_ESM.jpeg]

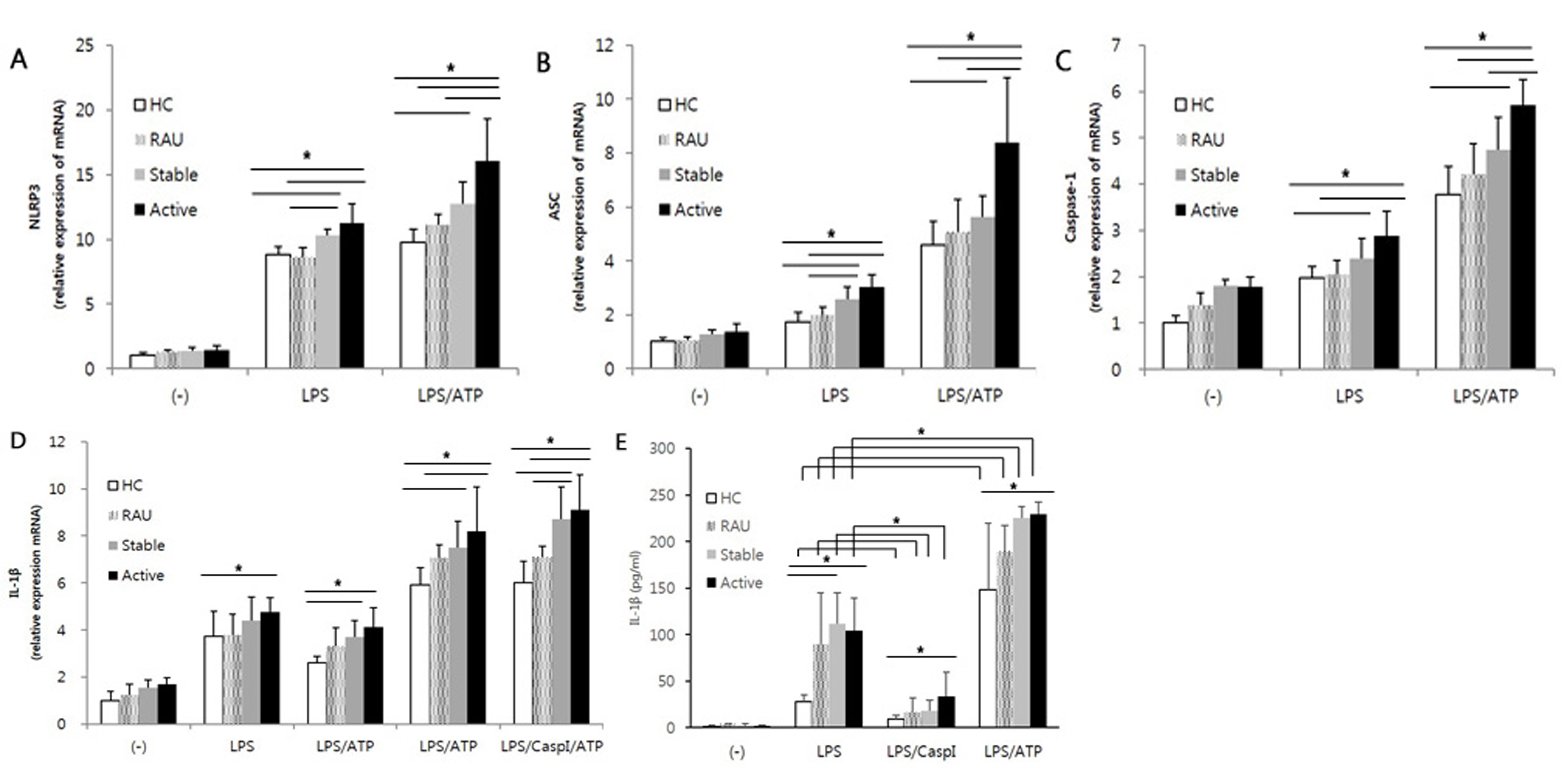

Supplement: Additional file 4: — The induced expression of NLRP3, ASC, caspase-1 and IL-1β is increased in Behçet’s disease (BD) compared to healthy control or recurrent apthous ulcer patients (RAU). PBMCs were initially stimulated for 4 h with LPS (100 ng/ml) with or without 20 μM zYVAD(Ome)-FMK, an irreversible caspase-1 inhibitor (LPS/CaspI). After 4 h, ATP (1 mM) was added to the cells for another 15 min (LPS/ATP or LPS/ATP/CaspI). (A-D) The mRNA expression of NLRP3, ASC, caspase-1 and IL-1β was measured by real time quantitative RT-PCR and normalized against the expression levels of glyceraldehyde 3-phosphate-dehydrogenase. The relative values are shown as a fold change to HC with no treatment (n = 8 per group). (E) Total IL-1β (n = 15 per group) was quantitated in the supernatant of stimulated PBMCs by ELISA. Data are represented as mean ± S.D. (*p < 0.05), (−): no treatment; LPS: lipopolysaccharide; ATP: adenosine 5-triphosphate; CaspI: caspase-1 inhibitor; HC: healthy volunteers, RAU: Recurrent apthous ulcer (disease control). [file 12950_2015_86_MOESM4_ESM.jpeg]
