# Supplementary material for: Increased expression of the NLRP3 inflammasome components in patients with Behçet’s disease
Source: J Inflamm (Lond). 2015 Jul 2;12:41. doi: 10.1186/s12950-015-0086-z (PMC4487834; doi:10.1186/s12950-015-0086-z)
Supplement: Additional file 2: — Detailed method for Quantitative real time PCR and Western blotting. [file 12950_2015_86_MOESM2_ESM.docx]

**Quantitative real time PCR**

Peripheral blood mononuclear cells (PBMCs) were prepared from heparinized blood samples by Ficoll Hypaque density gradients (Ficoll paque^TIM^ plus, StemCell Technologies, Vancouver, BC, Canada). PBMCs were stimulated for 4 hours with 100 ng/ml lipopolysaccharide (LPS; Sigma-Aldrich). After 4 hours, RPMI containing 1 mM adenosine 5-triphosphate (ATP; Sigma-Aldrich) was added to the cells for another 15 minutes (LPS/ATP). In separate experiments, 20 uM zYVAD(Ome)-FMK an irreversible caspase-1 inhibitor (CaspI; Enzo life science, PlymouthMeeting, PA) was added.

Total RNA was extracted using Trizol^®^ (Invitrogen, Carlsbad, CA, USA) according to the manufacturer’s instructions. Reverse transcription of RNA was performed using dNTP and oligo(dT) primers (Invitrogen, Carlsbad, CA, USA) and using Superscript™ III (Invitrogen, Carlsbad, CA, USA) following the recommendations of the manufacturer. Real-time PCR analysis was performed using primers and internal probes for NLRP1 (Hs00248187_m1), NLRP3 (Hs00918082_m1), ASC (Hs00203118_m1), Caspase-1 (Hs00354836_m1), IL-1β (Hs01555410_m1) and GAPDH (Hs99999905_m1), which were purchased as Assays on Demand primer-probe (Applied biosystems, Seoul, Korea). Glyceraldehyde-3-phosphate dehydrogenase (GAPDH) was used as the reference gene to normalize the RNA expression levels. Real-time PCR was performed on ABI Prism 7000 Sequence Detection System (Applied Biosystems, Foster, CA, USA) using TaqMan® Gene Expression Assays (Applied Biosystems, Foster, CA, USA) according to the manufacturer’s instructions. Subsequently, a Ct value was obtained for each sample. Quantification was done using the 2^−ΔΔCt^ method. The ΔCt values were calculated for each gene of interest as follows: ∆Ct_sample_ = (Ct_sample_ − Ct_GAPDH_) Relative mRNA levels were calculated by the expression 2^–∆∆Ct^, where ∆∆Ct = (∆Ct_sample_ − ∆Ct_calibration_). The calibrator sample (∆Ct _calibration_) was assigned from the HC group.

**Quantifying western blots**

Western blots were quantified using ImageJ analysis. The images were set to grayscale 8-type bit and a rectangular box was drawn to enclose a single lane. The box was then selected as the first lane and the same process was repeated for the remaining of the lanes. The relative density of the contents of each lane was then plotted as histograms and a straight line was drawn underneath where the peak ends to subtract any background. The wand tool was then used to get the measurements of each profile. Bands were normalized to β-actin by dividing the average intensity of the band by the average intensity of the β-actin band from the same sample labeled on the same gel.
